# White striping degree assessment using computer vision system and consumer acceptance test

**DOI:** 10.5713/ajas.18.0504

**Published:** 2018-11-28

**Authors:** Talita Kato, Saulo Martiello Mastelini, Gabriel Fillipe Centini Campos, Ana Paula Ayub da Costa Barbon, Sandra Helena Prudencio, Massami Shimokomaki, Adriana Lourenço Soares, Sylvio Barbon

**Affiliations:** 1 Department of Food Science and Technology, State University of Londrina (UEL), Campus Universitário, Londrina PR 86057-970, Brazil; 2 Department of Computer Science, State University of Londrina (UEL), Campus Universitário, Londrina PR 86057-970, Brazil; 3 Department of Animal Science, State University of Londrina (UEL), Campus Universitário, Londrina PR 86057-970, Brazil

**Keywords:** Appearance, Broiler Breast Fillet, Classification, Digital Image, Sensory Analysis

## Abstract

**Objective:**

The objective of this study was to evaluate three different degrees of white striping (WS) addressing their automatic assessment and customer acceptance. The WS classification was performed based on a computer vision system (CVS), exploring different machine learning (ML) algorithms and the most important image features. Moreover, it was verified by consumer acceptance and purchase intent.

**Methods:**

The samples for image analysis were classified by trained specialists, according to severity degrees regarding visual and firmness aspects. Samples were obtained with a digital camera, and 25 features were extracted from these images. ML algorithms were applied aiming to induce a model capable of classifying the samples into three severity degrees. In addition, two sensory analyses were performed: 75 samples properly grilled were used for the first sensory test, and 9 photos for the second. All tests were performed using a 10-cm hybrid hedonic scale (acceptance test) and a 5-point scale (purchase intention).

**Results:**

The information gain metric ranked 13 attributes. However, just one type of image feature was not enough to describe the phenomenon. The classification models support vector machine, fuzzy-W, and random forest showed the best results with similar general accuracy (86.4%). The worst performance was obtained by multilayer perceptron (70.9%) with the high error rate in normal (NORM) sample predictions. The sensory analysis of acceptance verified that WS myopathy negatively affects the texture of the broiler breast fillets when grilled and the appearance attribute of the raw samples, which influenced the purchase intention scores of raw samples.

**Conclusion:**

The proposed system has proved to be adequate (fast and accurate) for the classification of WS samples. The sensory analysis of acceptance showed that WS myopathy negatively affects the tenderness of the broiler breast fillets when grilled, while the appearance attribute of the raw samples eventually influenced purchase intentions.

## INTRODUCTION

White striping (WS) has been causing concern to the poultry industry recently, due to meat quality effects, leading to American consumers’ rejection owing to the product appearance [1]. However, in Brazil there are few sensory studies on the subject. And it is important to know the consumer’s assessment for these meats. WS is characterised by the appearance of white striations on the pectoralis major muscle, which follow the direction of the muscle fiber [1,2]. The etiology of WS is still unknown, and there are several contradictions about the quality of these meats [2]. WS classification [1,2] is based on the degree of WS partitioning: normal (NORM), moderate (MOD), or severe (SEV). However, this visual classification is subjective, in other words, each person can underestimate the size or amount of striations in the sample surface. Recently, new techniques have been used to evaluate the meat quality in a quickly and reliably way [3]. Image analysis has gained interest in analytical chemistry applications due to its simplicity, low cost and speed of response [4,5].

Besides that, CVS have been applied in several meat industry applications to eliminate the human bias during the visual evaluation [6]. Many of CVS apply machine learning (ML) techniques to simulate human decision taking. In fact, ML methods have been widely used for food classification and evaluation of spoilage, frauds, color, or to determine the most relevant parameters to assess meat quality [7–10].

These approaches can handle multiple parameters (such image’s attributes), are faster and accurate, do not degrade the meat samples and have low costs [11]. Several techniques have been used to classification tasks, but some standout, e.g. random forest (RF), multilayer perceptron (MLP), support vector machine (SVM), fuzzy rule-based systems (FRBS) and others [12]. Humans use visual aspects of meat to classify it in a determined striation degree. Developing an automated machine vision application to simulate this action requires the explanation of scene characteristics in some measurable quantity. It could be done by image features extraction. Many properties can be extracted from an image, like colour, pixels values distribution, statistical greatness, and frequency domain measures. They are used to characterise image appearance, allowing the application of ML algorithms to learn patterns and classify the meat samples. The poultry processing industry could benefit from a fast and precise system that can evaluate the poultry breast by image and classify the samples according to the quantity and distribution of striations, replacing the current subjective evaluation of WS assessment. To develop an application for striations segmentation is not a trivial task due to the small size and complex morphology of them. However, the texture of WS over meat could be explained using features calculated from the image, allowing the application of ML algorithms to learn patterns related to each degree of striation. In this research, the digital images of meat samples were processed, enhanced, segmented, and over them, features were extracted to describe the WS pattern. These metrics were used to induce the classification models based on the specialist vote. The objective of this study was to determine the efficacy of using a CVS to classify broiler breast fillets in three degrees of WS. And evaluate the consumer response on these samples. Moreover, the paper aims to investigate the most adequate ML approach (RF, MLP, SVM, and fuzzy) for WS degree assessment, simulating the human reasoning and choice, and the best set of image attributes to explain the WS phenomena variations. And finally, verify the consumer acceptance and purchase intent for broiler breast fillets with different degrees of WS.

## MATERIALS AND METHODS

### Data samples and trained panelist assessment

For the present study, 20 poultry breast fillets for each degree of severity (mixed sex, 47 days old) (total of 60 samples), were collected and transported under refrigeration to the laboratory for further analysis. These samples followed the conventional steps of slaughter (hanging, electrical stunning, bleeding, scalding, defeathering, evisceration, cooling in chiller, deboning and refrigeration) in a commercial line in Southern Brazil.

The samples were evaluated in three severity degrees [1,2] These degrees were: NORM (no striping on the surface), MOD (white lines, parallel to the muscle fibers with <1 mm thick but easily visible on surface) and SEV (white lines, parallel to muscle fibers, which were generally >1 mm thick and very visible on the fillet surface). In addition to a visual classification, a tactile evaluation was made to remove possible doubts among MOD and SEV degrees. SEV samples showed a decrease in tenderness when compared to MOD samples. This study was approved by the Ethics Committee and Animal Welfare (CEUA/UEL, the home institution - Protocol: 3159.2016.00).

The samples were digitalized using a digital single-lens reflex camera, model Nikon D7000 (CMOS sensor, 16.2 megapixels, 4.928×3.262 pixels). The camera was placed above the samples, at a distance of about 37 cm. For each meat sample, two photographs were taken aiming to choose the best capture. All camera parameters were set to automatic configuration. In this manner, our dataset was made up of a total of 120 images. Some images refer to the same sample and were used to validate image feature ability to classify the samples in a degree of WS.

### Software tools

Matlab was used to perform image processing routines for segmentation and to extract features from images. All the classification models were induced in R platform, with the RF, kernlab, RSNNS, and FRBS packages for RF, SVM, artificial neural network, and FRBS techniques, respectively.

In addition, the caret package was used to optimize the hyper-parameter choice during the model induction, and the FSelector package to choose the best subset obtained from image features.

### General overview of the approach

**Figure 1:**
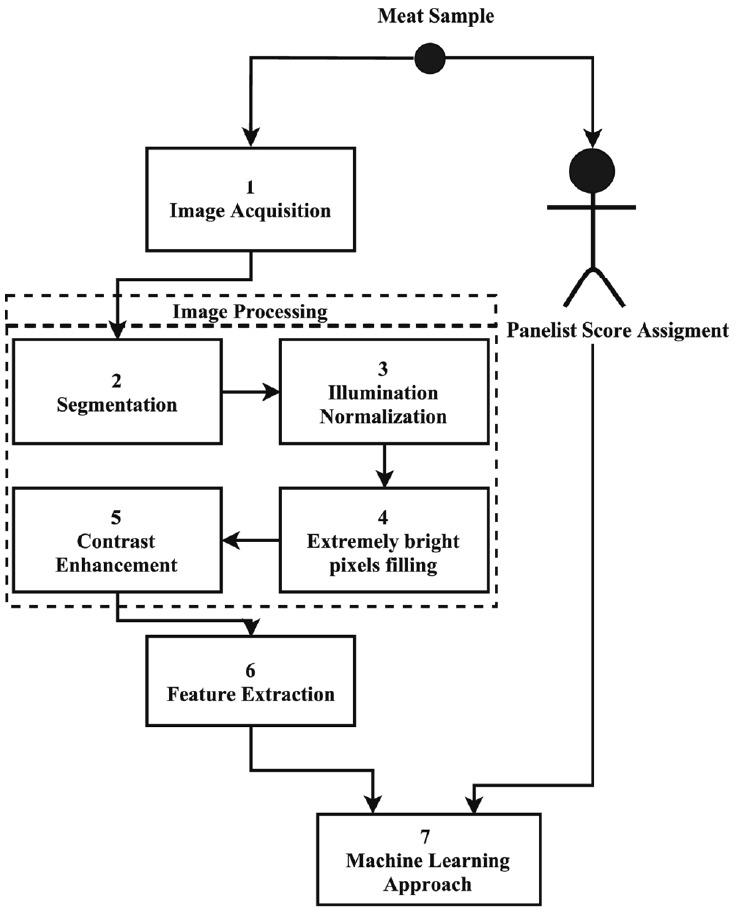
Overview of the proposed system.

Figure 1 presents an overview of our proposed system. The first step after meat sample WS assessment (by a trained panelist) was image acquisition (Step 1). The digital image was segmented (Step 2), and an illumination normalization process was performed (Step 3). The extremely bright pixels in the meat region, which were removed in segmentation (Step 2), were filled with information of neighbor pixels (Step 4). After, the image was subjected to a contrast enhancement technique in order to highlight the WS properties (Step 5).

From the resulting image, 25 features were extracted (Step 6), aiming to describe the WS behavior over meat surface. The most relevant subset of image features was investigated by a feature selection algorithm in these experiments. Using the panelist score, four supervised ML algorithms were tested (Step 7): RF, SVM, MLP, and a FRBS. The mentioned steps of the proposed system will be explained in details in the following sections.

### Image acquisition

**Figure 2:**
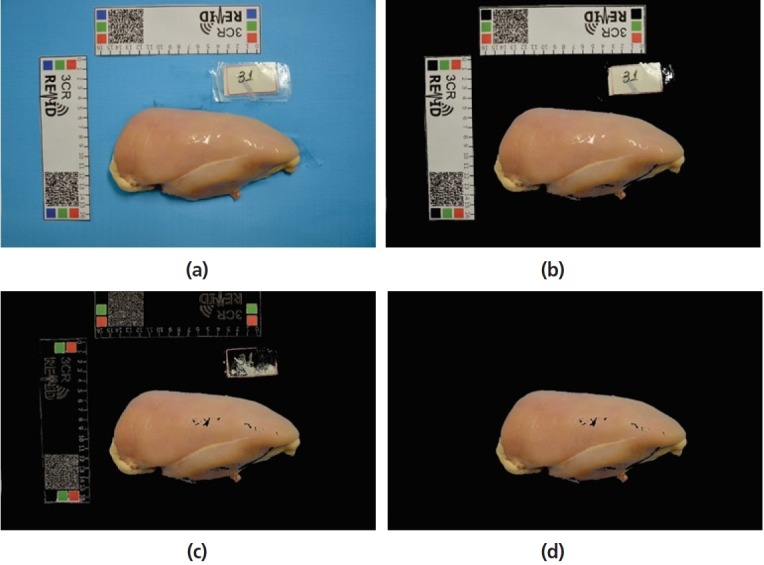
Image results from each step of meat segmentation approach. (a) original image; (b) background subtraction; (c) extremely bright pixel removal; (d) small region removal.

A common digital camera was used to capture the images. A blue paper was used as a standard background for image acquisition, aiming to get better contrast between the region of interest and other objects. In Figure 2, it is possible to observe white rulers near the meat sample so that they can offer further information about size and color calibration values [5].

Due to the illumination normalization step, which will be described in section 2.6, the environment illumination was not controlled for photographing. In fact, the room environment light was used, consisting of fluorescent lamps and solar light. With the adoption of this approach, it was possible to minimize the effects of heterogeneous incident light over the regions of interest.

### Segmentation

To accomplish image feature extraction from meat samples, firstly, it was necessary to segment the region of interest. Then, the segmentation step (number 1 in Figure 1) started by background subtraction. It was done using a threshold on H channel from hue, saturation, and value color space. The threshold could be obtained using Otsu’s technique, since it is one of the most accurate and widely used methods for image segmentation [13]. A sample image before background subtraction is presented in Figure 2a. In Figure 2b, the result for background subtraction is shown.

Extremely bright pixels correspond to high intensities in each R, G, and channels. These pixels are related to bright spots over the meat sample, a result of the surface reflex and the white rulers in the scene. They could be removed by a threshold value of each RGB channel, since they are not regions of interest for analysis. Removing pixels was the choice, since they had greater intensities than the average value of each channel.

Figure 2c shows the resulting image after the bright pixel removal. Some remaining non-meat areas were removed using a region growing algorithm. A binary mask was created with only two values: white, representing the meat, and black, representing other regions of the scene. Based on the size of image, small regions in mask were removed using a connectivity approach. After these processing steps, only the meat portion was kept in the image. Figure 2d presents the resulting image after small region removal and binary mask match.

### Illumination normalization

After the segmentation step, the image illumination was normalized [5], aiming to attenuate the effect of incident light spots. This method starts with a Gaussian blur filtering over an original image copy. It spreads the light spots increasing their radius, creating a gradient of intensity starting at the spot center.

The negative of the blurred image is taken so that the aforementioned spots become darker. The processed image is converted to the HSL color space to get only the reversed lightness information (the L channel). The L channel intensities can be combined with the original image to attenuate the lighter regions. An overlay blend operation between the reversed lightness representation and the original image results in an illumination normalized image. The overlay blend is given by the equation:

$$\text{E}=\frac{I}{255}\left(1+\frac{2\times M}{255}\times (255-I)\right)$$

**Figure 3:**
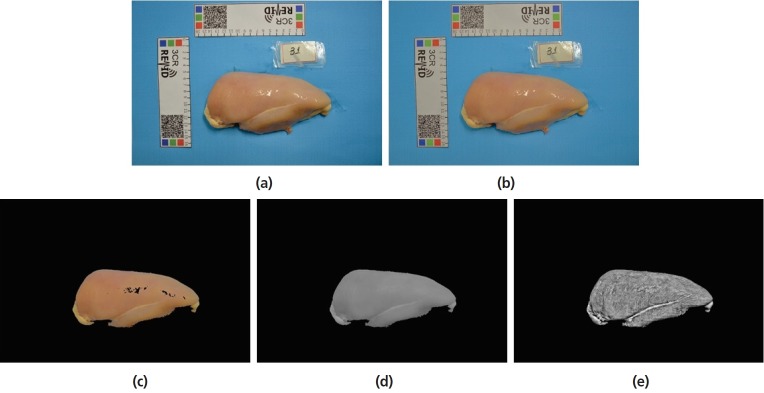
Image processing steps of proposed approach. (a) original image; (b) illumination normalization; (c) meat mask match; (d) extremely bright pixels filling; (e) contrast enhancement.

Where E is the resulting image, I is the lower layer (the original image) and M the upper layer (the described lightness image). As a result, dark regions become darker and light regions become lighter, according to the L channel values. Based on the reversed lightness image, the light spots are attenuated and the regions with homogeneous illumination are less changed. The illumination normalization effect is shown in Figure 3b.

### Meat region of extremely bright pixels filling

As it can be seen in Figure 2d, the resulting segmented region has some holes. They correspond to extremely bright pixels removed during the meat segmentation. The resulting black regions in the sample image may compromise the image features extraction, like texture descriptors, since these areas may be counted as a repetition pattern over meat surface. Therefore, a correction was applied to fill the aforementioned regions with the neighborhood pixel intensity information.

First, the image was converted to grayscale representation. Using the meat mask obtained from segmentation step, each black pixel inside the meat region was set to the average value of non-zero neighbor pixels. The pixel neighborhood was calculated using a square window with parametrized size. For the images used in the experiments, we set window size = 25×25 pixels. Figure 3d shows a meat sample with filled extremely bright pixels.

### Contrast enhancement

The normalized and filled image was subjected to the contrast limited adaptive histogram equalization (CLAHE) [14]. This technique was performed to increase contrast between meat region and WS. CLAHE technique has two parameters: Window size, corresponding to the length of blocks to subdivide the image for equalization, and the clip limit, which sets a limit on contrast enhancement. For images with 4,928×3,262 of resolution, we suggest: window size = (64×64) and clip limit = 0.07. Figure 3 summarizes the image processing steps of our approach. The original image is presented in Figure 3a–e show the result of illumination normalization, meat mask match, extremely bright pixel filling, and contrast enhancement, respectively.

### Feature extraction

Image features provide useful information for automatic classification [15]. In this paper, a set of 25 image features was explored. It consisted of histogram-based values [16], contrast and quality [17] descriptors, gray level co-occurrence matrix [18] and fast fourier transform (FFT) spectrum domain features [19].

Histogram-based features describe the frequency distribution of pixel intensities over the image. Statistical measures (mean, median, variance, standard deviation, skewness, and kurtosis) and other metrics related to the pixel distribution were calculated. These other metrics included the largest and the smallest histogram peaks, and the amount of non-zero values. The main contribution of histogram-based features is related to the aspects of tonal distribution without addressing spatial location and value concentration.

Contrast and image quality metrics are related to visual aspects of the image. They describe the perceiving level of separability between objects in a scene. The WS degree could be explained, among other aspects, through the variation of visual intensity between striations and muscle surface.

Gray level co-occurrence matrix stands for repetition patterns over the analyzed image. The periodicity of pixel neighborhood is calculated to describe the perceptual surface texture over the image. Based on the co-occurrence matrix, it is possible to detect patterns with a high level of repeatability. The WS occurrence might be described regarding repetitions of thin regions (striations), over a homogeneous surface (meat).

The FFT can be applied to change the perspective of image analysis. Instead of using a spatial domain, where a pixel has a *xy* location and intensity, a spectrum domain (frequency) representation is obtained. This representation allows the detection of periodic patterns in images by finding narrow peaks of high energy in the spectrum domain. A complete list of all image features used in experiments is presented in Table 1.

### Machine learning approaches

A classification task consists of an input vector describing a characteristic and deciding which of the N classes the data belong to. It is a supervised learning process, based on examples of N classes [20].

In a classification task, each instance (example) belongs to a class, and the set of classes covers the possible output domain. Sometimes an example might belong partially to more than one class, in this case, fuzzy classifiers should be used. In addition, a classification algorithm aims for generalization: produce sensible outputs for inputs not used in the training.

There are various classification methods. However, in general, all have the same objective: find decision boundaries to separate different classes. Some of these methods were briefly described below [21]. In this work, the performance of RF, MLP, SVM, and FRBS are compared. These algorithms were used for modelling classifiers used to analyze the WS degree based on features extracted from poultry meat images.

The RF method is an ensemble learning approach [22]. The main idea is the combination of many decision trees into a forest, naming the technique. Every tree inside the forest is built independently without pruning, by a subset of features. These features are chosen at random from all feature sets. The user must select the number of attributes used in each node and the number of trees inside the forest. Each tree in the forest uses a different training set, consisting of random sampling using a Bagging approach [22].

The MLP feedforward network is an important class of neural networks [23]. Usually, a network consists of three main components: an input layer, one or more hidden layers and, an output layer. The first layer receives the input values, which are computed in the nodes of hidden layer(s), giving an answer at the output layer. Information propagates through the network in the forward direction. The back-propagation [23] algorithm is often used to train the MLP networks. MLPs have been used to solve complex and diverse problems due to the generality of their application [23].

The SVM is a method of the ML [24], belonging to the class of kernel based methods. The main idea is to find the best hyperplane to separate data into determined classes. Therefore, SVMs aim to maximize separability, finding the instances of the problem that lie at the margin between the classes. The described values are called support vectors. SVMs are widely used in ML tasks due to their high accuracy, flexibility, and capacity to deal with high dimensional data [25].

The FRBSs are methods within soft computing, based on fuzzy concepts [26]. These methods are effective tools to deal with problems such as uncertainty, imprecision, and non-linearity. FRBSs are an extension of classical rule-based systems. They are mainly based on rules in the form “If A then B”, where A and B are fuzzy sets. In these experiments, the fuzzy-W technique, which is based on Ishibuchi’s strategy [27], was applied.

### Evaluation metrics

The information gain (IG) method was applied to reduce the problem of dimensionality by considering the original 25 features. This metric measures the feature capability to separate samples into classes of problem [28]. High IG value means more separation capability, in other words, a more important feature.

Each described classification model was run 50 times, using different train and test dataset configuration. A stratified holdout approach with 70% of the dataset for training was used. To evaluate the performance of the built classification models, accuracy, error rate, precision, and recall, the F-score was used.

Accuracy refers to the overall system performance. It is defined as the number of instances that were correctly classified among all cases. The error rate is related to the rate of misclassified samples, and it is defined as 1-accuracy. Precision is defined as the rate of true positives of a class *X*, among all instances classified as *X*. It shows the class agreement of the data labels with positive labels given by the predictor. Recall or sensitivity measures a classifier effectiveness to identify positive labels (classes). For each class, recall is defined as the number of true positive predicted cases, divided by the number of instances that belongs to the referred class. At last, the F-score measures the relation between data positive labels and those given by the predictor.

### Sensory analysis

Two sensory analyses were performed. The study was approved by the Human Research Ethics Committee (from Londrina State University, 1.842.109, CAAE 61901316.8.0000.5231). The three severities of WS were evaluated in random order and sequential presentation. In order to determine whether WS characteristics affected the sensory of broiler breast fillets, acceptance and purchase intention tests were performed differently in two days to present the samples.

The first sensory analysis was performed with broiler breast fillets purchased from a commercial establishment (same brand, lot) in Londrina-PR city. The samples were visually classified into three degrees of WS severity [1,2]: NORM, MOD, and SEV, totaling 75 samples (25 samples from each WS degree). This test was performed at the Sensory Analysis Laboratory of the Londrina State University. For this analysis, each broiler breast fillet (about 100 g) was prepared with 1.80% sodium chloride and was grilled with 2 mL oil at 140°C until samples reached 72°C at 75°C internally (measured with spit thermometer with digital display). After this step, fillets were cut (15 g), served in white disposable plates properly coded with 3 random digits. Consumers were placed in individual cabins, where they received instructions on the use of the scales.

For this test, 102 untrained judges were invited, all habitual broiler meat consumers. The consumer group consisted of 46.15% males and 53.85% females. In these groups, 95.19% are undergraduate students, who consume broiler meat 1 to 3 times a week (67.31%).

Consumers were asked to try the samples and evaluate them individually through a card using the 10 cm hybrid scale (0 = dislike extremely; 10 cm = like extremely). Finally, consumers evaluated their purchase intention regarding broiler breast fillets by using a 5-point structured scale (1 = certainly would not buy to 5 = certainly would buy). And they were also asked to explain what they liked or disliked about each fillet, in case they wanted to do so.

The second sensory test applied was the acceptance and purchase intention, that took place at the Comtour mall in the city of Londrina-PR, in front of a supermarket. One hundred and five untrained judges, who consume broiler meat regularly were clarified regarding the sensory tests. Subsequently, they received color photos of broiler breast fillets in actual sizes (photographed individually on foam trays used for commercialization, with a digital camera Sony Cyber Shot DSC-S950 10.1 mega pixels) in three degrees of WS in a random order (coded with 3 random digits). Evaluators consisted of 40.0% males and 60.0% females; ranging 36 to 50 years old (52.38%), most of them with elementary education (59.05%) and who consume broiler meat from 1 to 3 times a week (53.33%).

In total, 9 photos were prepared for this analysis (3 samples from each WS degree). And participants answered using the same method of evaluation mentioned: 10-cm hybrid hedonic scale (for acceptance test) and 5-point scale (for purchase intention). They were also asked to explain what they liked or disliked about each fillet, in case they wanted to do so.

### Statistical analysis

The results of all analysis were evaluated by analysis of variance and comparison of Tukey test averages at 5% probability (p≤ 0.05) among the three WS classifications using the Statistica 7.0 (StatSoft, Tulsa, OH, USA) program.

## RESULTS AND DISCUSSION

Considering the results of the proposed approach, it is possible to claim that our CVS can classify WS degree with considerable accuracy (86%). Table 2 summarizes the results obtained during the experiments for each ML algorithm. The techniques were sorted from the best to the worst using algorithm accuracy. It is possible to perceive that SVM, fuzzy-W, and RF obtained very similar general accuracy, about 86.4%. The worst performance was achieved by MLP (70.9%), which was related to the high error rate when predicted NORM meat samples. In fact, in these experiments it was determined that MLP was almost not able to handle with NORM degree samples, reflecting the strictly lower values obtained for precision, recall, and F-score.

**Figure 4:**
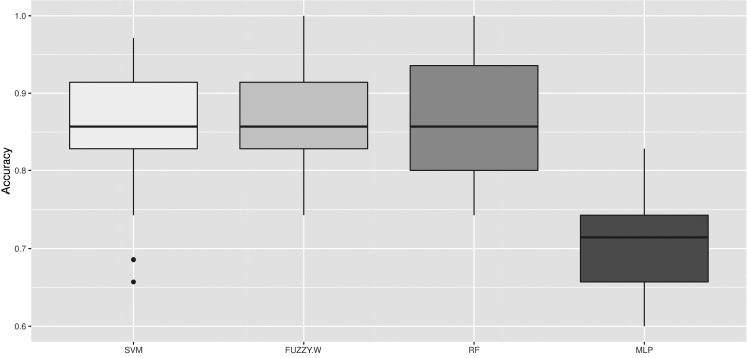
Boxplot of accuracies from models induced with SVM, fuzzy-W, RF, MLP, ML algorithms in classification task of poultry meat WS degree among NORM, MOD, and SEV.

Considering the three best techniques, RF and SVM presented greater standard deviation values (7.06% and 7.27%, respectively) when compared to fuzzy-W (6.89%). As it can be seen in the accuracy boxplot (Figure 4), SVM presented some outliers below the first quartile. RF had no outliers but showed the widest box, an indication of performance variability during training and test repetitions. The outliers obtained for SVM are an indication of instability of this method, depending on the train and test set configuration. In some cases, fuzzy-W and RF obtained 100% of accuracy, however, fuzzy-W presented the narrowest box, which indicates that this method was the most stable and robust technique for WS poultry meat degree prediction.

**Figure 5:**
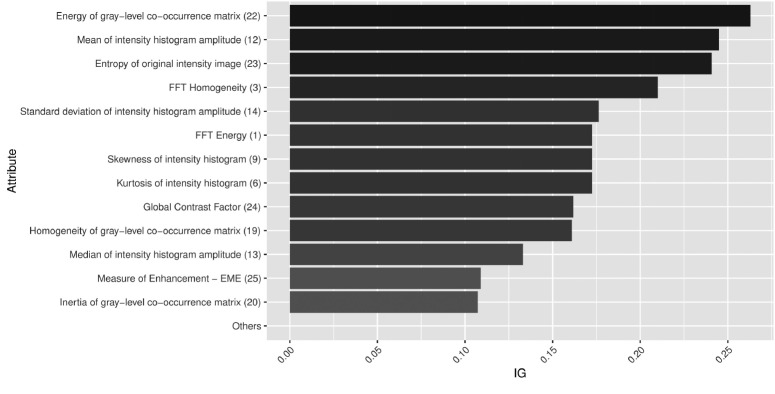
Importance ranking of image features with information gain (IG) metric for WS degree assessment.

Concerning the importance of specific image feature to assess the WS degree, it was possible to rank them by IG. Out of the original set of 25 features, only 13 attributes were relevant for classifying the WS degree. The IG of other attributes was equal to zero, thus they were not used to train the ML models. Figure 5 presents the ranking of image feature importance.

Feature 22, 12, 23, and 3 obtained the highest values of importance (IG>0.2). Feature 22 is a texture attribute extracted from gray level co-occurrence matrix, and describes the squared sum of co-occurrence matrix elements. In other words, it measures intensity pattern repeatability, like striations. Feature 12 is a histogram-based feature, measuring the mean intensity amplitude of the image. The striations are brighter than meat, so it could be a separating factor to determine the WS degree. The separability of striations and meat can also be explained through image entropy (feature 23), a statistical measure of randomness that can be used to characterize texture and contrast. At last, FFT homogeneity (feature 3) is another metric used to describe texture.

With less importance, features 14, 1, 9, 6, 24, 19, 13, 25, and 20 belong to different groups of image features. This result shows that just one type of image feature is not enough to deal with the WS degree assessment task. The remaining image features—features 2 (FFT entropy), 4 (FFT inertia), 5 (amount of non-zero groups in intensity histogram), 7 (peak of the largest non-zero group in intensity histogram), 8 (peak of the smallest non-zero group in intensity histogram), 10 (length of the largest non-zero group in intensity histogram), 11 (peak of the smallest non-zero group in intensity histogram), 15 (variance of the intensity histogram amplitude), 16 (amount of non-zero values on intensity histogram), 17 (peak of intensity histogram), 18 (entropy of gray level co-occurrence matrix), and 21 (correlation of gray-level co-occurrence matrix)—offered no contribution to describing WS degrees. They belong to different groups of image features, which reinforces the fact that just one technique for feature extraction is not powerful enough to describe all visual characteristics of WS phenomena.

### Hits and misses

**Figure 6:**
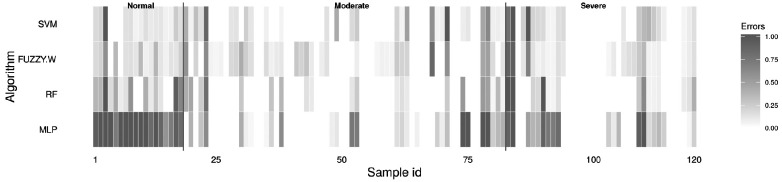
Accumulated misclassification predictions over 50 executions for classification of poultry meat samples in normal, moderate, and severe WS degrees. The error rate starts from 0 (no error) to 1 (error in all tests) illustrated from white to black color.

Figure 6 demonstrates the sample-by-sample accumulated error rate, once the error rate starts from 0 (no error) to 1 (error in all tests) illustrated from white to black color. The measure of errors was based on the ratio of misclassification to all determinations. As evidenced in Table 2, the lowest performance was achieved by MLP. This approach misclassified NORM degree samples in almost all cases, as shown in Figure 6. Despite that, SVM, fuzzy-W and RF obtained low accumulated error. Depending on training set combination, some pattern is obfuscated, decreasing the generalization capability. However, some samples presented high error rate for all classification algorithms. These samples correspond to the dark vertical aligned cells present in Figure 6.

Table 3 summarizes four of most misclassified images: 3, 23, 83, and 84. These images were misclassified by all algorithms in more than 40 executions. Image 3, which is related to meat sample 4, was classified as NORM by the trained panelist, and SEV by the ML techniques. Image 23 (sample 34) was predicted as SEV, and it was MOD. Similarly, images 83 (sample 25) and 84 (sample 42) were predicted as SEV by the panelist, and MOD by the ML techniques.

**Figure 7:**
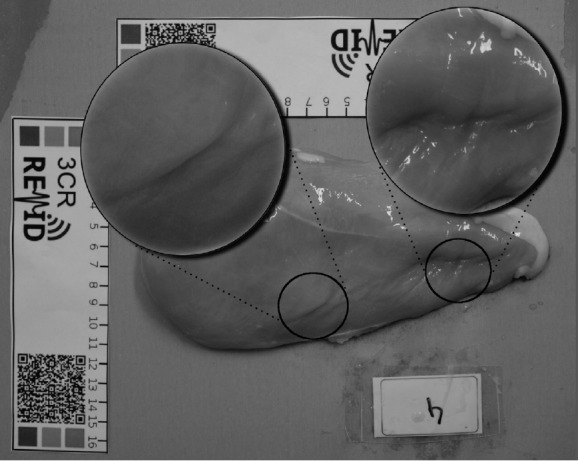
Misclassification of NORM as SEV in the image 3 (sample 4), a NORM sample misclassified by all ML techniques as SEV. Irregular surfaces in meat are highlighted and zoomed.

The visual distinction between NORM and SEV degrees is more pronounced than NORM and MOD, or MOD and SEV cases. In this way, misclassifying NORM as SEV is more impactful for the proposed WS assessment solution. This fact occurred in the classification of image 3. In order to diagnose the error, the features and the original image (Figure 7) were investigated. In Figure 7, it is possible to observe some regions with an irregular surface in muscle, indicating a lack of sample preparation for image acquisition. These regions are highlighted and zoomed in Figure 7, and present a very similar pattern of striations, as described by image features.

**Figure 8:**
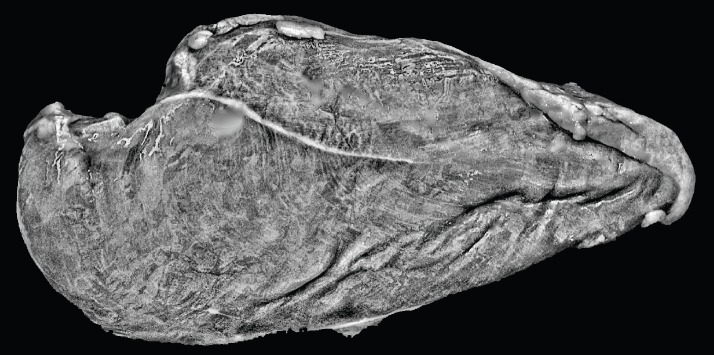
Image 3 (sample 4) after imaging processing steps: folds in meat were accentuated by the contrast enhancement technique.

Figure 8 presents image 3 after all the previously described image processing steps. The irregular surface of the meat was accentuated by the contrast enhancement technique towards very similar features to WS with SEV degree. It can be seen that the image feature values for this image were similar to SEV samples, leading to misclassification. Indeed, the meat sample preparation for image capturing is a major step of a CV approach. A lack of control may disrupt the striation visual characterization, decreasing the trained predictive modeling capability.

The misclassification variation for the other cases was of one degree. The visual characteristics of WS close degrees were very similar. Hence, the obtained error could be related to samples lying in inter-class separation margin. Besides, sometimes during WS level assessment, the specialist touched the samples to check its firmness. This occurred in some special cases, when only the visual classification caused uncertainty about the degree. SEV samples have a decrease in firmness when compared to MOD samples. This extra information is not related to visual aspects of meat and cannot be addressed by computer vision.

### Sensory analysis

The scores for the acceptance test and purchase intention of the grilled broiler breast fillets can be observed in Table 4. According to this study, it was evidenced that all hedonic grades of the acceptance attributes were between 7 and 8, indicating that, despite the classifications, all degrees of WS were accepted in appearance, aroma, flavor, texture and global acceptance.

Among the analyzed attributes, only texture showed significant differences (p<0.05) between the classifications analyzed. Samples classified as SEV were the only ones that presented significant differences when compared to the others. Other researchers [29], showed higher values for WS samples classified as SEV in cooking loss results (26.74%), due to loss of liquids resulting from protein denaturation during cooking. This information could have influenced our sensory results, since the samples were prepared on the grill, influencing the final texture of the broiler breast fillets. The appearance of the samples did not present significant differences, as they passed through the grill that eventually camouflaged the characteristics of WS. They are predominantly located on the surface of the fillets.

Broiler breast fillets classified as SEV [30], presented significant differences in the texture sensory descriptive analysis. The hardness attribute was the only one that differed the samples classified as SEV from the others. The same authors [30], also attributed this greater effort to chewing the sample with the increase of the connective tissue content in the WS fillets.

With the use of broiler breast fillets photos for the sensory analysis, all the hedonic scores of the appearance attribute were between 3 and 7 (Table 5), indicating a great variation in the values, unlike the previous sensory analysis for the same attribute. In this second sensory test, samples classified as SEV obtained the lowest scores for the appearance attribute and presented significant differences (p<0.05) between the other WS degrees.

The photos, differently from the previous sensory test, had the objective of analyzing only the appearance attribute to simulate the products found in commercial establishments. According to the research comments, 20% of consumers were able to identify the presence of white lines characteristic of WS, and even without knowing its composition, they would reject the product by its appearance. Furthermore, 30% of consumers, in addition to identifying a presence of white striation, observed a fat-like surface deposition.

These comments were verified in the purchase intention notes. All means of the different classifications differed from each other. The highest mean was presented for the samples classified as NORM, MOD, SEV, respectively. Therefore, with the increase in the WS samples severity degree, the notes of acceptance and purchase intention tests decrease.

These results were similar to other study [1], in which American consumers also evaluated photographs of samples in the three degrees of WS severity, through images selected and viewed through computers. These researchers concluded that the presence of striations (increasing of WS severity), decreases consumer acceptability of broiler breast fillets. According to this American research, more than 50% of consumers reported they probably would not/definitely not buy the broiler breast fillets with any degree of white striation.

## CONCLUSION

CVS proved to be accurate, fast and robust for the classification of WS meat. The classification models SVM, fuzzy-W and RF showed the best results with similar general accuracy (86.4%). The worst performance was obtained by MLP (70.9%) with the high error rate in NORM sample predictions. The sensory analysis of acceptance verified that WS myopathy negatively affects the texture of the broiler breast fillets when grilled and the appearance attribute of the raw samples, which influenced the purchase intention scores of raw samples. Therefore, the characteristics of WS samples could economically damage the sales of broiler breast fillets. The results were satisfactory, although further studies with physical-chemical analysis are needed to validate image analysis results and to implement this system in a real processing industry in a production line with a greater number of samples.

## Figures and Tables

**Table 1:** List of all image features extracted

No.	Type	Description
1	FFT	FFT energy
2	FFT	FFT entropy
3	FFT	FFT homogeneity
4	FFT	FFT inertia
5	Inten Hist	Amount of non-zero groups in intensity histogram
6	Inten Hist	Kurtosis of intensity histogram
7	Inten Hist	Peak of the largest non-zero group in intensity histogram
8	Inten Hist	Peak of the smallest non-zero group in intensity histogram
9	Inten Hist	Skewness of intensity histogram
10	Inten Hist	Length of the largest non-zero group in intensity histogram
11	Inten Hist	Length of the smallest non-zero group in intensity histogram
12	Inten Hist	Mean of intensity histogram amplitude
13	Inten Hist	Median of intensity histogram amplitude
14	Inten Hist	Standard deviation of intensity histogram amplitude
15	Inten Hist	Variance of intensity histogram amplitude
16	Inten Hist	Amount of non-zero values on intensity histogram
17	Inten Hist	Peak of intensity histogram
18	Co Matrix	Entropy of gray-level co-occurrence matrix
19	Co Matrix	Homogeneity of gray-level co-occurrence matrix
20	Co Matrix	Inertia of gray-level co-occurrence matrix
21	Co Matrix	Correlation of gray-level co-occurrence matrix
22	CoMatrix	Energy of gray-level co-occurrence matrix
23	Contrast	Entropy of original intensity image
24	Contrast	Global contrast factor
25	Image quality	Measure of enhancement (EME)

**Table 2:** General results summary of WS degree prediction, ordered by accuracy

Algorithm	Accuracy		Error		Precision		Recall		F-Score	
				
	Mean	STD	Mean	STD	Mean	STD	Mean	STD	Mean	STD
SVM	0.8646	0.0706	0.1354	0.0706	0.8554	0.0852	0.8585	0.0805	0.8460	0.0828
Fuzzy-W	0.8640	0.0689	0.1360	0.0689	0.8530	0.0874	0.8619	0.0801	0.8417	0.0841
RF	0.8640	0.0727	0.1360	0.0727	0.8210	0.1061	0.8644	0.0924	0.8325	0.1000
MLP	0.7097	0.0552	0.2903	0.0552	0.5641	0.0517	0.6251	0.1142	0.6973	0.1097

STD, standard deviation; SVM, support vector machine; RF, random forest; MLP, multilayer perceptron.

**Table 3:** Samples with higher misclassifications rate

Image	Panelist	SVM	Fuzzy-W	RF	MLP
3	NORM	SEV	SEV	SEV	SEV
23	MOD	SEV	SEV	SEV	SEV
83	SEV	MOD	MOD	MOD	MOD
84	SEV	MOD	MOD	MOD	MOD

SVM, support vector machine; RF, random forest; MLP, multilayer perceptron; NORM, normal; SEV, severe; MOD, moderate.

**Table 4:** Acceptance of sensory attributes and purchase intention of WS broiler breast fillets

Degree	Appearance	Aroma	Flavor	Tenderness	Overall acceptance	Purchase intention
NORM	8.10±1.75	7.83±1.88	7.97±1.95	8.19^a^±1.77	8.09±1.74	4.08±0.96
MOD	8.13±1.65	7.63±2.16	7.54±1.72	7.73^a^±1.81	7.67±1.55	3.80±1.00
SEV	7.63±1.87	7.89±1.82	7.68±2.08	7.47^b^±1.83	7.64±1.83	3.85±1.08

NORM, normal; MOD, moderate; SEV, severe.

a–b Means within a column followed by different superscript letters differ significantly (p≤0.05).

**Table 5:** Acceptance of appearance and purchase intention of WS broiler breast fillets photos

Degrees of WS	Appearance	Purchase intention
NORM	7.32^a^±1.67	4.00^a^±0.61
MOD	6.94^a^±1.76	3.31^b^±0.85
SEV	3.20^b^±2.12	2.04^c^±1.16

NORM, normal; MOD, moderate; SEV, severe.

a–c Means within a column followed by different superscript letters differ significantly (p≤0.05).
